# Evaluation of physical activity programmes for elderly people - a descriptive study using the EFQM' criteria

**DOI:** 10.1186/1471-2458-11-123

**Published:** 2011-02-21

**Authors:** Ana I Marques, Maria J Rosa, Pedro Soares, Rute Santos, Jorge Mota, Joana Carvalho

**Affiliations:** 1 Research Centre in Physical Activity, Health and Leisure - Faculty of Sports, Porto University, Portugal; 2 Department of Economics, Management and Industrial Engineering - University of Aveiro, Portugal; 3 Escola Sec. José Estêvão - Aveiro, Portugal; 4 Maia Institute of Higher Education (CIDESD), Portugal

## Abstract

**Background:**

In the past years, there has been a growing concern in designing physical activity (PA) programmes for elderly people, because evidence suggests that such health promotion interventions may reduce the deleterious effects of the ageing process. Quality is an important issue when designing a PA programme for older people. Some studies support the Excellence Model of the European Foundation for Quality Management (EFQM) as an operational framework for evaluating the quality of an organization. Within this context, the aim of this study was to characterize the quality management models of the PA programmes developed by Portuguese Local Administration to enhance quality of life for elderly people, according to the criteria of the EFQM Excellence Model.

**Methods:**

A methodological triangulation was conducted in 26 PA programmes using questionnaire surveys, semi-structured interviews and document analysis. We used standard approaches to the statistical analysis of data including frequencies and percentages for the categorical data.

**Results:**

Results showed that Processes (65,38%), Leadership (61,03%), Customer results (58,46) and People (51,28%) had high percentage occurrences of quality practices. In contrast, Partnerships and resources (45,77%), People results (41,03%), Policy and strategy (37,91%), Key performance results (19,23%) and Society results (19,23%) had lower percentage occurrences.

**Conclusions:**

Our findings suggest that although there are some good practices in PA programmes, there are still relevant areas that require improvement.

## Background

The last few decades have witnessed a significant demographic ageing process, causing deep social and political transformations, and challenging society and humanity's options for the 21st century. The population aged 60 or over is increasing rapidly and is expected to increase by more than 50 per cent over the next four decades, expanding from 264 million in 2009 to 416 million in 2050 in more developed regions [1]. Subsequently, there will be more older people than children in the world population for the first time in history.

The most important issue related to demographic ageing deals with its implications for the well-being of the elderly, such as access to appropriate health-care services. In developed countries, some degree of progress has been made to achieve this objective, all the more so as ageing is the most important contributor to the increase in health care costs [2].

The concept of *'active ageing' *has been employed by the World Health Organization (WHO) since the late 1990s, and is defined as '*the process of optimizing opportunities for health, participation and security in order to enhance quality of life as people age*' (WHO 2002 [3] p.12). Therefore, there has been a growing concern in designing physical activity (PA) programmes for elderly people, since evidence indicates that such health promotion interventions may reduce the deleterious effects of the ageing process [4,5] and improve quality of life [4-7]. Nevertheless, a substantial proportion of European elderly people have lower PA levels than those recommended for good health [8,9]. Therefore, increasing adherence to PA among elderly people is an important public health challenge.

The Centers for Disease Control and Prevention (CDC) developed guidelines with other American organizations for increasing PA across a large number of settings and populations, including elderly people [10]. They described a set of recommendations and strategies to improve programmes, developing new approaches and highlighting the need for effective programme evaluation [11,12]. This *'imperative' *has a wide application (CDC 2002b [13] p.5) that reveals commitment to provide high quality programmes. Furthermore, programme evaluation is a useful tool for continuous quality improvement [14] and the WHO guidelines for the evaluation of health promotion emphasize the need to evaluate and propose the allocation of adequate resources for this action [15].

*Healthy Ageing - A Challenge for Europe Report *[16] suggests a systematic application of quality management/assurance methods to increase project's quality; these indicate that Quality is an important issue for PA programmes for older people.

**Figure 1:**
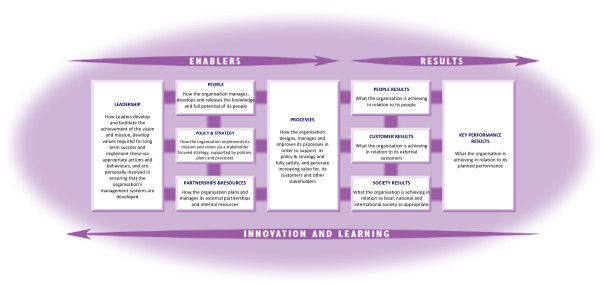
**EFQM Excellence Model (adapted from EFQM, 2003)**.

With the purpose of helping organizations to improve their quality, the European Foundation for Quality Management (EFQM) introduced the EFQM Excellence Model in 1991 with the support of EOQ, the European Organization for Quality, and the European Commission. The EFQM Excellence Model is a non-prescriptive framework based on nine criteria divided into thirty-two sub-criteria [17]. Of these nine criteria, five are 'Enablers' - what an organization does to achieve excellence - and four are 'Results' - what an organization achieves, i.e., the results achieved on the path to Excellence. As illustrated in Figure 1, the arrows presented in the Model show its dynamic nature; the issues related to 'Innovation and Learning', while horizontal vectors essential to the Model's architecture, also emerge as cross-sectional elements in all the criteria. They show innovation and learning can improve 'Enablers', which in turn lead to improved 'Results'. The Model recognizes that there are many approaches to achieving sustainable Excellence in all aspects of performance, based on the premise that: *"Excellent results with respect to Performance, Customers, People and Society are achieved through Leadership driving Policy and Strategy that is delivered through People, Partnerships and Resources, and Processes" *(EFQM 2003a [17] p.5).

The application of the EFQM Excellence Model promotes the use of a management methodology based on objective criteria that is applicable to all areas of business and constitutes a self-assessment exercise of the organization's quality. Self-assessment will shed light on the areas requiring improvement, as well as on the process and actions necessary to conduct improvement. The Model is currently used by thousands of organizations throughout Europe, such as firms, health institutions, schools, public safety services and governmental institutions, among others. It provides organizations with common management terminology and tools, thus facilitating the sharing of best practices between organizations of different sectors [18].

Despite the numerous PA programmes for the elderly that have been created in recent years - especially by the Public Local Administration - their evaluation is scarce. Moreover, the EFQM Excellence Model had never been used in PA programmes for elderly people.

In this context, the purpose of this study was to characterise the quality management models of the PA programmes developed by the Portuguese Local Administration to enhance quality of life for elderly people, according to the criteria of the EFQM Excellence Model 2003.

## Methods

### Procedures

In order to gather empirical evidence, methodological triangulation -- i.e. questionnaire surveys, semi-structured interviews and additional document analysis -- was employed.

A preliminary on-line questionnaire was sent out to all mainland Portuguese municipalities (n = 278) in May of 2008. This brief questionnaire provided the following information: geographic localization, name and objectives of PA programmes, age of the PA programme, characteristics of age groups and participants' age, number of activities included in the PA programme, frequency of the programme (days/week), quality initiatives, organization name and the identification details of the PA programme's coordinator (Additional file 1).

Of the 278 municipalities, a total of 97 valid questionnaires were answered. Since some municipalities provided more than a single programme, 125 PA programmes were identified. Inclusion criteria for the purposive sample implied that at least one of the following conditions should be verified: i) programmes should belong to a District Capital in order to apply a geographic criterion; ii) programmes should include the following cumulative criteria: a) must have been in practice for 10 years or more [19], b) must have had two or more different types of activities [20,21], and c) must have had a frequency of two or more times a week [6]; iii) programmes that apply a quality initiative [14,16,22-25]. Therefore, 27 potentially eligible PA programmes for elderly people were identified, of which 18 were from a District Capital; eight were aged ten years or more, had two or more types of activities and a frequency of two or more times a week; and one had a quality initiative (Quality Certification). We screened each PA programme's coordinator by telephone to check eligibility, confirm willingness to participate and, accordingly, provide a written informed consent by email. At this stage, one programme was excluded because it did not meet any of the three conditions above. The characteristics of the 26 PA programmes included in our sample are described in Table 1.

**Table 1:** Characteristics of the 26 PA programmes

id	Age (years)	Minimum/maximum age to enrol	Participants' average age	Number of activities	Frequency (days/week)	Quality initiatives	Organization
A	[1; 5[	55 years/90 years	71	1	1	no	Municipal Government
B	[1; 5[	55 years/no limit	72	2	4 or +	no	Municipal Government
C	[5; 10[	55 years/80 years	65	4 or +	4 or +	no	Municipal Government
D	[5; 10[	55 years/no limit	70	2	2	no	Municipal Government
E	[5; 10[	55 years/no limit	71	4 or +	4 or +	no	Municipal Government
F	[1; 5[	55 years/80 years	65	1	2	no	Municipal enterprises of sport
G	[5; 10[	60 years/no limit	69	4 or +	2	no	Municipal enterprises of sport
H	[5; 10[	55 years/no limit	71	4 or +	4 or +	no	Municipal enterprises of sport
I	[1; 5[	55 years/80 years	66	3	2	no	Municipal enterprises of sport
J	[1; 5[	60 years/no limit	71	2	2	no	Municipal Government
K	[1; 5[	55 years/no limit	68	4 or +	2	no	Municipal Government
L	≥10	60 years/no limit	72	4 or +	4 or +	no	Municipal Government
M	≥10	65 years/no limit	71	2	4 or +	no	Municipal enterprises of sport
N	[5; 10[	55 years/no limit	68	4 or +	3	yes	Municipal Government
O	≥10	55 years/90 years	72	4 or +	4 or +	no	Municipal Government
P	≥10	60 years/no limit	70	4 or +	3	no	Municipal Government
Q	[5; 10[	55 years/no limit	71	2	4 or +	no	Municipal Government
R	[5; 10[	55 years/no limit	69	3	4 or +	no	Municipal Government
S	[5; 10[	65 years/no limit	70	2	2	no	Municipal Government
T	[1; 5[	55 years/no limit	65	3	4 or +	no	Senior University (Municipal)
U	≥10	55 years/no limit	71	4 or +	2	no	Municipal Government
V	[1; 5[	65 years/no limit	70	2	2	no	Municipal Government
W	≥10	55 years/90 years	70	1	3	no	Municipal Government
X	≥10	60 years/no limit	72	2	2	no	Municipal Government
Y	≥10	60 years/no limit	71	4 or +	4 or +	no	Municipal Government
Z	[1; 5[	55 years/no limit	65	2	4 or +	no	Municipal Government

To characterise the quality management models of the PA programmes, *semi-structured face-to-face interviews *with the PA programmes' coordinators (n = 26) were carried out between February and April of 2009. The questions were based on the EFQM Excellence Model's nine criteria and 32 sub-criteria. Before the 26 interviews, a pilot study was conducted among four PA programmes' coordinators, conveniently chosen from among the programmes that were not selected for the sample, to understand the process and evaluate the content understanding of the questions. As a result, some questions were adapted in accordance with respondents' comments. Afterwards, a standard interview guide was created and used for all interviews, which lasted 45 to 60 minutes and were tape-recorded and transcribed *verbatim *at a later date. Participants were asked about each sub-criterion of Leadership, Policy and Strategy, People, Partnerships and Resources, Processes, Customer Results, People Results, Society Results and Key Performance Results. A content analysis of the transcribed interviews was conducted. Two coding strategies were applied: (a) a *priori *categorisation of data based on the 32 sub-criteria and (b) a *posteriori *coding scheme, obtained directly from the data, using an inductive method to identify the themes and subthemes that emerged. To ensure rigour and reliability of analysis, the first three transcripts were coded in their entirety by two coders who achieved agreement through discussion and consensus. Two independent researchers double-coded two transcripts to assess the inter-rater reliability of coding. Intra-rater reliability was also conducted on a question of each criterion, within a 5-day interval. The inter-rater and intra-rater reliability were assured by the intercoder and intracoders' agreement, from Bellack's formula [26]. Both results obtained ranged from 95% to 100%, confirmed by Cohen's Kappa to eliminate the agreement by chance. Interscore reliability was in the range of 0.93 and above. To facilitate the coding process, we used the QSR NVivo software, which helps manage and organize qualitative data.

An *on-line questionnaire *was also administrated to the 26 PA programmes' coordinators, between June and July 2009. This new questionnaire, based on the EFQM Excellence Model's nine criteria and 32 sub-criteria, was generated according to the literature review and the interviews' content analysis. For each sub-criterion, items were devised concerning the areas addressing the EFQM Excellence Model and the specificity of the PA programmes for elderly people. Closed questions with multiple choice answers and Likert scales were used. The first draft of the questionnaire was submitted to a panel of experts (n = 5) in the field of PA programmes for elderly people and/or EFQM Excellence Model, to ensure the content validity. The experts pointed out their level of accordance with the relevance of the items, ease of understanding and adequacy as an instrument to characterise the management models of the PA programmes. Based on their suggestion, fourteen items were reframed and two were eliminated, due to its irrelevance. After, the on-line questionnaire was tested among 15 PA programmes' coordinators, chosen from among the programmes that were not selected for the sample, for comments on readability. Some adjustments were made to make the questions clearer and more relevant to the PA programme case. The study design also included a test-retest reliability of the answers, performed with an interval of seven days. Agreement was estimated using kappa statistics (κ for categorical variables) and weighted kappa statistics (κw for ordinal variables). High levels of agreement (0.86 to 0.97) were found. The final version of the on-line questionnaire comprised 165 items and took a respondent about one hour to complete.

In addition, *document analysis *was carried out. Written documents, including procedures, budgets, flyers, e-mails, reports, minutes of meetings, specifications, print screens, publications, price lists, etc. were made available by some of the coordinators. Other information was gathered from the web page of the organization.

We used standard approaches to statistical analysis of data including frequencies and percentages for the categorical data, performed with the Statistical Package SPSS, version 17.0.

### Data presentation

A set of the most relevant items concerning quality practices associated with the EFQM Excellence Model criteria was adapted from an original scale created to measure the nine criteria [27] and assigned to each EFQM sub-criterion based on its content domain. Several adjustments were made to reflect the specificity of the PA programmes for elderly people, according to collected data. The presence or absence of a particular quality practice was encoded as: addressed/measured = 1; not addressed/not measured = 0.

## Results

Regarding *Leadership*, most of the coordinators who participated in this study revealed that they were personally involved in the development of a culture of Excellence, reinforcing a strong communicative culture throughout all areas of the organization (84,62%), encouraging people's empowerment and autonomy and ensuring that every member of the organization knows the role that the PA programme should play in society (both with 80,77%). Almost two-fifths (38,46%) of the coordinators ensured that people were capable of taking initiatives and fulfilling their responsibilities in the most appropriate way, and a single leader collaborated in quality training since only his programme was involved in a quality scheme (3,85%) (Table 2).

**Table 2:** Frequencies and percentages of quality practices in the criterion Leadership

1. Leadership	n	%
1a. Leaders develop the mission, vision, values and ethics and are role models for a culture of Excellence		
Coordinators encourage people to feel empowerment and autonomy	21	80,77
Coordinators participate and give support to continuous improvement processes	19	73,08
Coordinators collaborate in quality training by teaching people at lower hierarchical levels	1	3,85
Coordinators ensure that all members of the organization have a clear idea of what the PA programme's position should have in society	21	80,77
1b. Leaders are personally involved in ensuring the PA programme management system is developed, implemented and continuously improved		
Coordinators become involved in running the PA programme as a set of interrelated processes, all for achieving quality	14	53,85
Coordinators ensure that people are capable of taking initiatives and assimilating better ways of doing their responsibilities	10	38,46
1c. Leaders interact with customers, partners and representatives of society		
Coordinators take part in continuous improvement processes, even when these activities go beyond Coordinators' responsibilities	16	61,54
Satisfaction of current and future customers ensures the success of the PA programme	16	61,54
To improve in a particular aspect, coordinators and other members of the organization collaborate with other organizations with PA programmes to help each other	15	57,69
1d. Leaders reinforce a culture of excellence with the organization's people		
There is a strong communicative culture throughout all areas of the organization	22	84,62
The involvement of people can only be achieved if coordinators are the first to show commitment, practicing what they preach	14	53,85
Coordinators behave in a way that allows the integration and mobilization of members of a team	18	69,23
1e. Leaders identify and champion organizational change		
Coordinators stimulate the continuous improvement of services and processes	19	73,08
Coordinators continuously acquire and update knowledge that is valuable for the PA programme	16	61,54
Coordinators act in a way that makes it easier for people to accept proposed changes voluntarily	16	61,54

Concerning *Policy and Strategy*, the issues related to quality initiatives, such as the measurement of quality and non-quality costs, quality strategies and quality objectives were referenced by one coordinator (3,85%), the one who's programme was involved in a quality initiative. In contrast, 84,62% of the coordinators reported the identification of organizational processes and their interrelationships and 80,77% stated that all people are familiar with the mission and objectives of the PA programme (Table 3).

**Table 3:** Frequencies and percentages of quality practices in the criterion Policy and Strategy

2. Policy and strategy	n	%
2a. Policy and strategy are based on the present and future needs and expectations of stakeholders		
The establishment of PA programme objectives takes people's opinions into account	15	57,69
The establishment of PA programme objectives takes external opinions into account	7	26,92
Effective management is based on information about customers	11	42,31
Customers' needs are taken into account when establishing objectives	11	42,31
2b. Policy and strategy are based on information from performance measurement, research, learning and external related activities		
Continuous improvement processes are based on a systematic assessment of PA programme effectiveness	16	61,54
Systematic measurement of quality and non-quality costs is carried out	1	3,85
Information systems are in place to capture external information (about customers, society...)	10	38,46
2c. Policy and Strategy are developed, reviewed and updated		
Systematic procedures are in place to plan, evaluate and control PA programme goal achievements	16	61,54
Quality strategies affect all organizational areas and coordination activities	1	3,85
Quality objectives stem from long-term strategic plans	1	3,85
Coordinators favour consensus about relevant objectives and future projects	5	19,23
2d. Policy and Strategy are communicated and deployed through a framework of key processes		
Organizational processes and their interrelationships are identified	22	84,62
Coordinators inform people about the quality strategy	1	3,85
Every member in the organization knows the PA programme mission and objectives	21	80,77

In relation to the criterion *People *(the same as employees/workers), 84,62% of the coordinators reported that People maintain fluid communication with one another; in contrast, 15,38% indicated that People voluntarily pass on useful information to other members of the organization. Two items related to quality initiatives appear with a diminutive percentage (3,85%), namely People's access to information about quality results and the quality training they are offered. The majority of the coordinators (80,77%) stated that formal processes were used to find out people's opinions (Table 4).

**Table 4:** Frequencies and percentages of quality practices in the criterion People

3. People	n	%
3a. People resources are planned, managed and improved		
Formal processes are used (such as attitude surveys or people briefing) to find out people's opinions	21	80,77
Emphasis is placed on recruiting highly skilled people	16	61,54
A higher level qualification, specifically related to PA and ageing, is required for instructors	9	34,62
3b. People's knowledge and competences are identified, developed and sustained		
Specific quality training is offered to people	1	3,85
People continuously update their skills in their specific area of knowledge	20	76,92
Staff members are provided with means for extensive training	10	38,46
3c. People are involved and empowered		
People are allowed to decide how the work is done	8	30,77
People's opinions are taken into account when defining PA programme objectives	20	76,92
People are given the opportunity to suggest and implement solutions to work problems	16	61,54
People's autonomy and participation are encouraged	13	50,00
Teamwork is a common practice	15	57,69
3d. People and the organization have a dialogue		
Formal communication channels are in place to provide information about customers' needs	18	69,23
Formal communication procedures are established with all stakeholders	20	76,92
People have access to information about quality results	1	3,85
People maintain fluid communication with one another, going beyond the formal structure of the organization	22	84,62
Internal communication is totally open and transparent	15	57,69
People voluntarily pass on useful information between one another	4	15,38
3e. People are rewarded, recognized and cared for		
Coordinators explicitly recognize people's achievements at work	11	42,31

With reference to *Partnerships and Resources*, less than 20% of the PA programmes had formal communication procedures with partners and 11,54% of coordinators revealed that relationships with academic partners allow the organization to have access to scientific information. Nearly three quarters (73%) of respondents reported that the organization has the capacity for external cooperation. The most reported item was the one related to the recording of information and knowledge (88,46%) (Table 5).

**Table 5:** Frequencies and percentages of quality practices in the criterion Partnerships and Resources

4. Partnerships and resources	n	%
4a. External partnerships are managed		
Cooperation with partners provides the organization with high quality of resources	8	30,77
Formal communication procedures are established with partners	5	19,23
Relationships with academic partners allow the organization to have access to scientific information	3	11,54
Relationships with health partners allow the organization to have access to health information	13	50,00
The organization has capacity for external cooperation	19	73,08
4b. Finances are managed	14	53,85
4c. Buildings, equipment and materials have a maintenance plan	9	34,62
4d. Technology is managed		
Technological innovations are implemented	18	69,23
4e. Information and knowledge are managed		
Systematic records are made	23	88,46
The latest scientific knowledge is pursued	7	26,92

Analysis of the *Processes *criterion showed the items recommendations concerning exercise sessions phases and standardized systems to deal with customer complaints were accomplished by all PA programmes. We can also verify that most of the organizations advertised the PA programme and good accessibility was guaranteed (96,15%). Nonetheless, just 30,77% of organizations were oriented towards the fulfilment of customers' expectations and needs and only 19,23% kept documentation of work methods and organizational processes (Table 6).

**Table 6:** Frequencies and percentages of quality practices in the criterion Processes

5. Processes	n	%
5a. Processes are systematically designed and managed		
Work methods and organizational process are explicitly defined	22	84,62
There is comprehensive documentation about work methods and organizational processes	5	19,23
Organizational processes are periodically revised	16	61,54
Work processes exist to promote efficient behaviour patterns throughout the organization	19	73,08
Emergency protocols are periodically revised	9	34,62
5b. Processes are improved, as needed, using innovation in order to fully satisfy and generate increasing value for customers and other stakeholders		
Development and innovation of processes is emphasized	12	46,15
5c. Services are designed and developed based on customer needs and expectations		
The organization knows which services customers need	18	69,23
The organization is oriented towards the fulfilment of customers' expectations and needs	8	30,77
5d. Services are produced, delivered and serviced		
The organization is committed to develop PA programmes for older adults, concerning the components: aerobic fitness, muscular-strength, balance and flexibility	17	65,38
Preparticipation screening is designed to guarantee the safe participation of customers	11	42,31
Recommendations about the components of the exercise training session are followed (warm-up, stretching, conditioning and cool down phases)	26	100,00
Progression in the exercise training sessions is followed	18	69,23
The front desk is the central point of contact between the organization and the customer	17	65,38
The organization advertises its services	25	96,15
Environmental conditions of exercise sessions are guaranteed	15	57,69
Good accessibilities to the PA programme are guaranteed (side-walks, passenger transportation)	25	96,15
Access to the programme are facilitated by different processes or pathways	20	76,92
5e. Customer relationships are managed and enhanced		
Standardized systems are in place to deal with customer complaints	26	100,00
Standardized systems are in place to deal with customer suggestions	14	53,85

Concerning *Customer results*, 76,92% of the programmes evaluated customers' satisfaction and 34,62% had measures and/or indicators of customers' loyalty (Table 7).

**Table 7:** Frequencies and percentages of quality practices in the criterion Customer Results

6. Customer results	n	%
The organization has measures and/or indicators of customers' satisfaction	20	76,92
The organization has measures and/or indicators of customers' loyalty	9	34,62
The organization has measures and/or indicators of the communication procedures with customer	14	53,85
The organization has measures and/or indicators of the complaint resolution procedure	18	69,23
The organization has measures and/or indicators of the customers' PA outcomes	15	57,69

Relating to *People results*, 69,23% of the programmes evaluated people's absenteeism and 15,38% had measures and/or indicators of people's organizational commitment (Table 8).

**Table 8:** Frequencies and percentages of quality practices in the criterion People Results

7. People results	n	%
7a. People motivation and commitment		
The organization has measures and/or indicators about people's willingness to work	8	30,77
The organization has measures and/or indicators about people's organizational commitment	4	15,38
7b. People achievement		
The organization has measures and/or indicators of the capability of people to identify work problems and to provide solutions	15	57,69
The organization has measures and/or indicators of how people share organizational values	6	23,08
The organization has measures and/or indicators about people's initiative	11	42,31
The organization has measures and/or indicators regarding people's performance (e.g. results of evaluations)	17	65,38
7c. People satisfaction		
The organization has measures and/or indicators of people's absenteeism	18	69,23
The organization has measures and/or indicators of people's loyalty	7	26,92
The organization has measures and/or indicators of people's satisfaction	10	38,46

Concerning *Society results*, 15,38% PA programmes had measures and/or indicators of their involvement in their target community. 23,07% of the coordinators confirmed that the organization had measures and/or indicators of the programme's impact in society (Table 9).

**Table 9:** Frequencies and percentages of quality practices in the criterion Society Results

8. Society results	n	%
The organization has measures and/or indicators of the programme's involvement in community	4	15,38
The organization has measures and/or indicators of the social responsibility of the programme	5	19,23
The organization has measures and/or indicators of the programme's impact in society (awards, media reports, invitations, etc...)	6	23,07

In *Key performance results*, one coordinator mentioned assessments of the quality of the service delivered and 42,31% of the coordinators reported that the organization has measures and/or indicators of the financial results of the PA programme (Table 10).

**Table 10:** Frequencies and percentages of quality practices in the criterion Key Performance Results

9. Key performance results	n	%
9a. Financial results		
The organization has measures and/or indicators of its financial results	11	42,31
9b. External results		
The organization has measures and/or indicators regarding the quality of the service delivered	1	3,85
The organization has measures and/or indicators regarding the partners management	5	19,23
9c. Results on processes		
The organization has measures and/or indicators of the process efficiency	3	11,53

**Figure 2:**
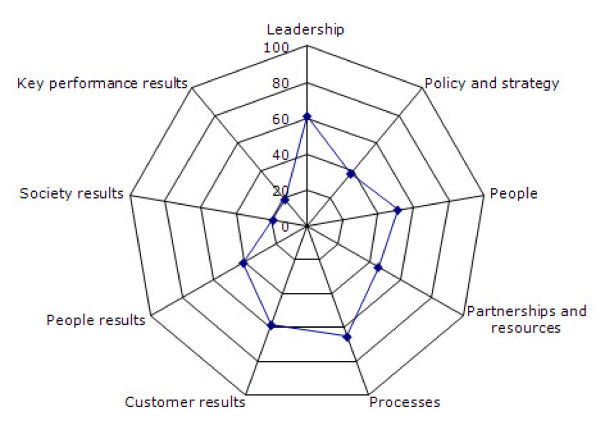
**Average of the percentages related to quality practices of the EFQM Excellence Model's criteria**.

Figure 2 shows the average of the percentages related to quality practices associated to the EFQM Excellence Model criteria. Four criteria (three Enablers and one Result) had values over 50%: *Processes *(65,38%), *Leadership *(61,03%), *Customer results *(58,46) and *People *(51,28%). In contrast, the other two Enablers and three Results had percentages under 50%: *Partnerships and resources *(45,77%), *People results *(41,03%), *Policy and strategy *(37,91%), *Key performance results *(19,23%) and *Society results *(19,23%).

## Discussion

To our knowledge, this was the first study applying the EFQM Excellence Model criteria to PA programmes for elderly people.

Results showed that Processes, Leadership, Customer results and People had high percentage occurrences of quality practices. In contrast, Partnerships and resources, People results, Policy and strategy, Key performance results and Society results had lower percentage occurrences.

PA programmes for elderly people play a significant role in senior citizens' health, quality of life, autonomy and capability to face daily tasks. It is widely accepted that the benefits of such programmes depend upon adherence to exercise [28]. Higher attendance in PA programmes and activity levels are strongly influenced by degrees of enjoyment [29,30]. Therefore, continuous quality improvement of the PA programmes for elderly people can be useful, and even critical, for elderly satisfaction and adherence.

*Leadership *is the key for driving forward quality improvement activities [31-33] and involves a process of social influence on a group of people. Our data suggests that the coordinators are particularly involved in developing the vision and mission, and enhance a strong culture of communication. These aspects are considered fundamental to quality management [34-36]. Indeed, other studies in different sectors have focused on leadership and have shown that the commitment of the leaders operates as the thrust of the quality improvement process [37-39]. Moreover, their physical presence, visibility and concern for quality improvement were associated with transformational leadership [40], i.e., leadership that creates valuable and positive change in its followers. Our study also revealed that most of the leaders interact with customers, partners and representatives of society. Trustworthy leadership increases partnership building and sustainability, essential to guarantee the success of PA promotion as a public health strategy, as demonstrated in some programmes [41]. Several studies have focused on customers [42-44] since listening them appears to be a priority for organizations that want to succeed. With regard to PA programmes, the CDC mention the importance of interacting with all stakeholders [13]. Specifically related to the PA programmes for elderly people, the British Heart Foundation (BHF) stated that participants or other stakeholders must be actively involved in all aspects of programme development, including planning, promotion and evaluation [45]. The ACSM also recognizes that PA leaders should work closely with individuals to design a PA regimen that reflects the person's preferences and capabilities [46]. In addition, our results indicate that coordinators neglect to run the PA programme as a set of interrelated processes. Although there are no studies on this issue for PA programmes for elderly people, some organizations have made recommendations for their specific programme, namely the American Association of Cardiovascular and Pulmonary Rehabilitation (AACVPR), which states that the programme leaders are responsible for directing, integrating and coordinating programme services, and recommending a central location for all policies, procedures and guidelines references [31]. Another interesting result of our data concerns the fact that most of the leaders are not involved in quality training in terms of teaching people at lower hierarchical levels, which might be related to the fact that only a single programme concerned itself with quality initiatives.

*Policy and strategy *is defined as how the organisation implements its mission and vision via a clear stakeholder-focused strategy, supported by relevant policies, plans, objectives, targets and processes [17]. Our results point out a modest concern about the opinions of different stakeholders in setting targets for the PA programme, which has been described as one of the crucial steps in the planning and evaluation of PA programmes, or as a good practice [13,45]. In addition, contrary to the guidelines [45], our study showed that a minority of programmes establish the objectives according to the participants' stated aims. Furthermore, this fact is in the opposite direction from the results of an European cross-national report on PA Programmes and promotion strategies for older people, in which most of the PA Programme's directors reported that their programmes were adjusted according to the participants' aims [19]. Another result that stands out in our data is the fact that just about two thirds of the programmes systematically assess their effectiveness in order to improve their continuous quality improvement process, which opposes the *Benchmark 3 *from Physical Activity and Health Branch (PAHB), at the CDC [14]. As indicated by the CDC, *'the evaluation is the systematic examination and assessment of features of an initiative and its effects, in order to produce information that can be used by those who have an interest in its improvement or effectiveness' *(CDC 2002b [13] p.5), consequently an '*imperative*', as stated before. Jackson argues that every effort must be made to engage the organisational members in continuous improvement activities [47]. However, no programme can be planned or evaluated oblivious of the context that surrounds it, especially when what drives most decisions on policy and practice in the public sector are considerations of the available evidence [45]. Institutional, community and public policies may have either supporting or antagonistic effects on programmes [48]. In addition, there are several factors that influence health behaviour [49]. Therefore, it is necessary to include pertinent information regarding the programme context [13,14] that must be absorbed in different ways [50]. In the present study, only 38,46% of PA programmes capture this information, which may reflect a limited knowledge on the part of most of the programmes about the context in which they operate. On the other hand, about two thirds of the analysed programmes have an annual plan that is regularly reviewed and used in an annual report. The data from this report helps to improve the new annual planning cycle of the PA programme. These procedures are in agreement with those found in other studies [51,52] or in accordance to different documents, such as content of the planning and evaluation of PA programmes [13,53] and health promotion programmes [54]. Still regarding this criterion, most of the leaders of our study reported that everybody had full access to the information about the mission and objectives of the PA programme. In the field of Higher Education, Calvo-Mora and collaborators [37] alleged that the leader's communication and involvement of all staff in policy and strategy were crucial to the processes management. Moreover, in accordance with the same author [37], our study found that processes were clearly identified, as well as their interrelationships. With regard to quality strategies, in our study only one PA programme had regularly used internal quality assessment and external audits. However, several studies have focused on the reasons for the use of quality schemes and pointed out the advantages of their implementation in improving services [24,55,56]. On the other hand, Ritchie and Dale suggest the existence of some obstacles to implementing these initiatives within the organizations [57]. Similarly, Davies and collaborators reviewed the aspects of culture/context, which were specific to the university academic context, and could impact negatively on the implementation of a quality framework [58].

Regarding *People *criterion, that is an important feature for quality management [59], most of the participants in our study reported the existence of procedures to find out employees' opinions, which was also found in a study related to quality management in sports facilities [60]. This initiative is considered a quality practice to Connolly and Connolly [61]. In fact, organizations have recognized the need to understand employee opinions to identify their concerns, assess the impact of a variety of agendas and provide employees with different communication channels [62]. Regarding this issue, our data also show that employees from the majority of PA programmes have an open dialogue with all stakeholders, especially with one another (76,92%). Furthermore, although the results are less obvious with regard to autonomy and decision-making, our study demonstrates that most of the PA programmes involved and empowered people in various ways (e.g. opinions and suggestions put forward by people, and teamwork). These findings are not totally in line with the arguments of Wilkinson and collaborators, who emphasized the employee involvement as a key theme for quality management, namely autonomy, creativity, active cooperation and self-control for employees [63]. Also, Osseo-Asare and collaborators concluded that a conceptual framework for achieving and sustaining quality in UK higher education institutions could be developed based on a set of principles which includes staff empowerment through participation and commitment [38]. In their study, these authors found a discrepancy between what respondents think about the importance of staff empowerment and the real practice in the organizations. Even with regard to the management of people, most of the participants in our study gave emphasis to the recruitment of people with high skills; however, only 34,62% require a specialization in the area of PA and ageing for instructors. These results are similar to those found on the *Cross-National Expert Survey Report on Physical Activity Programmes and Physical Activity Promotion Strategies for Older People *[19]. In this report, the authors make recommendations on the importance of recruiting teachers who have high levels of qualification and reinforce the importance of continuous professional development. Regarding this issue, the *International Curriculum Guidelines for Preparing Physical Activity Instructors of Older Adults *outlines each of the major content areas that should be included in any entry-level training programme [64]. The PAHB, established that a PA programme should be run by highly skilled PA practitioners [14]. Regarding the continuous training of people, our study revealed that over three quarters of the PA programmes take this aspect into account. In contrast, Hughes and collaborators found that only 56% of the PA programmes for older people trained their instructors [65]. The *Guidelines for Cardiac Rehabilitation and Secondary Prevention Programs *also emphasises these points, and goes further, establishing that the *'polices and procedures should include provisions for a competency-based job description; required education, continuing education, experiences, licences and certifications; and an orientation checklist, a competency assessment and a regularly performed - at least annually - performance appraisal' *(AACVPR 2004 [31] p.193). Once more, our data showed that the items related to quality initiatives have only a passing reference, which appears to be related to the fact that just a single programme is involved in quality schemes, as previously explained.

Different studies reported that the opportunities that are provided by *Partnerships and resources *should be maximized [38,60,66,67]. In addition, the development and sustainment of the community partnerships is the first public health benchmarks for PA Programmes established by the PAHB at the CDC [14]. In our study, 73,08% PA programmes have established partnerships, which is in line with the emphasis that some authors [41,68,69] have put on the importance of forging effective partnerships, creating value and promoting cooperation agreements based on mutually beneficial joint synergies. Especially in the PA programmes for elderly, some organizations reinforce the importance and strength of these partnerships, since they provide additional resources in the form of funding, facilities and equipment and being able to access wide-ranging abilities and knowledge [3,45]. The most surprising result of our data concerns the few partnerships with Higher Education Institutions (11,54%). Indeed, these academic institutions contribute to the creation of knowledge and its dissemination, so we consider it a disadvantage for programmes to not have direct access to their counsel. Moreover, such partnerships would have reciprocal benefits, since the programme also could provide means for researchers to get their answers in a more practical way. Additionally, disseminating this knowledge may promote the development of new programmes or improve the programme itself [13]. When we analyzed the partnerships with health institutions, the results are better, but still far from what is supported by some authors or organizations, who advocate the active participation of healthcare professionals in counselling patients on PA [45,70-72] or encouraging them to accumulate moderate-intensity PA [73]. Similar results arise from the European Network for Action on Ageing and Physical Activity (EUNAAPA) study, where sixty percent of the PA programme directors reported that they build partnerships with local healthcare professionals or organisations [19]. With regard to finances, our results appear to indicate that there is not a strict control of these resources, since there is still a considerable percentage of programmes that do not manage them (65,38%). These results are quite different from those reported by Scott and colleagues [19], where sixty five percent of the PA programme directors were able to estimate the total cost of their programme. In fact, most of the monetary funds of these programmes come from the public finance, and thus it appears to us that leaders should control these funds even more strictly. Although the PA programmes are not-for-profit, the management of its financial resources should be identified as key-process, in order to consolidate the programme's financial structure and to ensure it can fulfil its mission in the present and in the future. Despite the maintenance plans of equipment and buildings should be periodically provided [66], just about one third of the interviewed coordinators reported that their programme had maintenance plans. Another study [19] found a higher percentage of programmes with maintenance plans (46%), but the results were still not consistent with the recommendations [31,74]. Otherwise, the recognition that information technology has been a catalyst for progress and prosperity [75] seems to be accepted by the coordinators of our study, since most of them implemented new technologies in their programmes. Concerning information management, although there are no recommendations in the field of PA programmes for elderly, the AACVPR advises that information management involves supervision of the storage, communication, utilization and tracking of information related to the programme and facility [31]. In this respect, the majority of the coordinators indicated that information, concerning to all aspects of the programme, was systematically recorded. On the contrary, the results related to the systematic pursuit of the latest scientific knowledge are quite modest, since less than one third of the coordinators refer to this quality practice. The reason for this unexpected result becomes somewhat clearer when we realise that very few programmes have established partnerships with higher education experts who are up to date on the latest scientific knowledge. In an American study [76] most states provided evidence of competency with regard to using data and scientific information to develop and prioritise their PA programming.

An excellent organization adopts a management philosophy based on *Processes *[77,78]. Although the majority of the coordinators of our study stated that the methods and processes were defined, only a minority operationalised it in terms of documentation. For the AACVPR, policies and procedures related to information management should include a wide range of records and should specify uniform standards for evaluation, intervention and outcome measurement [31]. Furthermore, processes should be systematically reviewed [17,79]. Specifically with regard to emergency protocols, about one third of the coordinators stated that they are carried out periodically. Related results arise from the EUNAAPA study, where half of PA programme directors reported having emergency protocols in place and that staff members were trained annually, at the very least, in these protocols [19]. Both results indicate that AHA/ACSM's recommendations have not been followed. In fact, it is emphasized that emergency policies and procedures must be reviewed and practiced regularly [74]. With regard to the design of services and tailoring the programme to the needs and interest of participants, the results differ. On the one hand, more than two-thirds of coordinators recognized that the services are designed according to customer needs; on the other hand, less than a third is geared towards the fulfilment of their expectations and needs. In the Scott and collaborators study, almost two thirds of PA directors reported that participants were formally surveyed for the aims of their involvement in the programme and most of these directors also reported that their programmes were adjusted according to participants' stated aims [19]. Physical activity leaders should work closely with individuals to design a PA regimen that reflects the person's preferences and capabilities [46]. In the same line, the BHF recommends the involvement of participants in this process (BHF 2007). Moreover, tailoring the exercise programme to the needs and interest of participants is associated with higher programme attendance [80,81]. With regard to the preparticipation screening, less than half of our PA programmes' coordinators reported that a health check was required to guarantee a safe participation of the customers. Results from EUNAAPA study [19] are slightly different since only half of the PA programme directors reported that a health check was required before a potential participant would be eligible to enter their programme. Screening of older adults prior to starting an exercise programme continues to be a controversial issue [82]. In fact, the ACSM endorses the perspective that medical clearance should not be required prior to encouraging older individuals to begin a light-intensity activity programme, since it may be a disincentive to increasing PA among these individuals [46]. For higher intensity levels, AHA/ACSM recommend a pre-participation screening, primarily to identify those at increased risk of an adverse cardiac event [74]. In our study, about two-thirds of the PA coordinators indicated that the exercise prescription includes aerobic, muscle strength, flexibility and balance exercises. Additionally, they also reported incorporating progression as part of their programme. These are consistent with the ACSM position's stand [6] and ACSM's Guidelines [83]. In our study we found an unanimous result concerning the components of the exercise training session, which is in line with the ACSM recommendations [83]. Our results about exercise prescription, progression and components of the session are more consistent with the ACSM recommendations than those disclosed in the EUNAAPA study [19]. Concerning to environmental conditions, more than half of the coordinators reported that they are guaranteed, i.e. temperature of sports facilities, safe and pleasant conditions of sports equipment and facilities, places with good acoustics and access to a water source are incorporated in the programme. This represents an adequate degree of concordance with the recommendations [31,83]. With regard to advertising, more than three quarters of the coordinators revealed that the programme was promoted. Some authors and organizations believe that social marketing and communication campaigns are a part of a set of actions required to increase PA [12,84,85]. In addition, the BHF makes recommendations on marketing and promotion strategies among older people [45]; however, no scientific evidence was found about the most effective method of promoting a PA programme for this target population. Across all programmes, 76,92% offer different forms of access to facilitate the enrolment of seniors. The Task Force on Community Preventive Services recommends the creation of or enhanced access to places for PA, combined with informational outreach activities to increase PA [12], even giving examples of how to reduce some environmental barriers. Good accessibility is also provided in almost all analysed programmes (96,15%), which is an essential aspect of programme planning [12,45,72]. The BHF emphasises the proximity of programmes to residences in a friendly and accessible way, ensuring well-lit paths and providing good public transports [45]. In this regard, a qualitative study in older and rural African American and white women found that PA programmes' enabling factors included transportation and free facilities [86]. A study by Booth and collaborators showed that for adults over 60, neighbourhood safety and access to local facilities were important predictors of being active [87]. In our study, all the programmes had an effective complaints handling system and more than half had suggestions through standardized processes. In addition to what was mentioned above about the importance of customer suggestions or opinions, customer complaint information can be also used as a basis for customer-focused process improvement [88]. In this particular case, our results suggest that organizations have a preference for reactive methods and delayed methods, such as complaint analysis, over proactive methods, contrary to what was found in another study [44]. An excellent service can only be achieved with a profound knowledge of evolving customer needs; therefore, a functional customer complaint management system should be implemented in every organization [89].

With respect to *Customer results*, organizations must measure and achieve them [17]. Similarly, PA interventions should be evaluated in terms of their processes as well as their outcomes [11]. There are many studies addressing the measurement of PA in order to identify current levels of activity and assess the effectiveness of intervention programmes. However, few PA intervention studies specifically target Customer retention or Customer satisfaction. Actually, the EFQM argues that excellent organisations achieve the best results for their customers and achieve high levels of customer satisfaction [17]. Furthermore, customers do not only provide input (suggestions or complaints), but they also take part in the service process, influencing both the process's performance and the perception of quality of the service produced [90]. One of the most commonly used techniques for listening to customers is satisfaction surveys [44]. More than three quarters of our PA programmes' coordinators assured that the satisfaction of participants in their programme was formally measured. Another key predictor of customer results is loyalty [36], but less than 35% of the programmes studied evaluate this item. A recent study about PA programmes for older adults in the United States found that 74% tracked attendance [91]. Also, complaints handling and management are essential for achieving customer retention and loyalty [92]. Besides this, though all programmes have a complaints system in place, only approximately 70% evaluated their resolution process. Contrary to complaints, all the programmes that have a standardized system of suggestions also carried out its assessment. Although the measurement process represents one of the most important components of customer results from an exercise programme [83], just 57,69% of our coordinators reported that objective outcome measures were recorded for participants at regular intervals.

To achieve excellence, organisations must also focus on the *People results *[17], since people involvement is one of the most important drivers of continuous improvement [77]. Nevertheless, most coordinators of our study revealed that the organization does not have information on its employees' motivation and commitment. This result is not surprising, especially because organizations rarely use instruments to obtain information about how their employees assess the motivational aspects of their workplace [93], compared with job satisfaction measurement. However, some meta-analysis studies [94,95] concluded that people's satisfaction is not enough to improve their performance - people must also be highly motivated [93]. Furthermore, without satisfied and motivated employees it is impossible to achieve satisfied and loyal customers [44]. An empirical study observed that employees' loyalty is significantly related to service quality, which in turn impacts customer satisfaction and customer loyalty [96]. Martin-Castilla and Rodriguez-Ruiz give examples of the different aspects that must be evaluated, both in terms of people's motivation and satisfaction, such as the development of professional careers, learning opportunities, definition of objectives, employment conditions, salary, relation between peers, organisational role in the community, and work environment, among others [78]. Additionally, one of the key indicators of people satisfaction includes absenteeism [36]. While the majority of our PA programmes' coordinators confirmed that there were indicators of people's absenteeism (69,23%), only a minority stated that the employees' loyalty was measured (26,92%) as well as people's satisfaction (38,46%). We believe that people who are satisfied with regard to the management, employment conditions, relationships between peers and the organisational role in the community will be more prone to improve the quality of the PA programme; therefore, the evaluation of theses issues should not be neglected. Also, people's achievement is an important indicator, not only with regard to the development of people, but also in their ability to solve problems and take initiatives. Nearly two thirds of our PA coordinators had indicators of people's performance, which is defended by the AACVPR [31], as discussed previously. This result stems from the fact that the majority of people with employment contracts in the public sector is evaluated by the *Integrated System on the Evaluation of the Public Administration Performance *(SIADAP).

The *Society results *criterion is based on what an organisation is achieving in satisfying the needs and expectations of the community [17]. The programme's visibility, engagement and reputation are recognized as a result of its activities and the active participation of the organisation as a responsible member of the community. However, few participants (19,23%) reported indicators of the involvement of their programmes in the community and less than one quarter of the programme's impact on society (23,07%). Furthermore, the CDC claims the importance of assessing the programme effects on organizations or communities [13], but this is not our case. In fact, it is not just the impact of the programme from the standpoint of public health, but also the perceptions that society has about the programme as a barometer of its action in society. Also, social responsibility is a vital part of the work and role of the programme, as it tries to respond to a problem of the society as a whole [77], but again, only nearly 20% of the PA programmes' coordinators had measures or indicators to track this issue. As recognized by some authors [45,97], community involvement in these programmes is critical to its success, so it is concerning that the most of the coordinators do not pay attention to these indicators.

The *Key performance results *represent the global organizational performance and the fulfilment of expectations. The mission of the PA programmes is linked to a significant impact on the promotion of PA in the elderly population. However, less than 12% of our coordinators declared they had indicators of process efficiency, i.e. obtaining the best outcomes from a set of actions. Also, regarding the quality of the service delivered, only one PA coordinator assumed that this assessment was performed. This result may be associated with the fact that only one programme performed a quality assessment/audit. In this respect, several studies [23-25,55] found that quality initiatives may improve process and outcomes. Finally, less than fifty percent of the PA coordinators indicated that the organisation's financial resources were properly managed. Recognising that most of the PA programmes have limited municipal funds, we believe that there is still a modest understanding of the need to achieve a certain level of profitability to contribute to the sustainability of the programme, and that all activities must be cost-accountable.

The '*evaluation is integral to success' *(Schmid 2006 [11] p.115) so, regardless of sector, size, structure or maturity, organisations need to establish an appropriate management framework to be successful [98]. We believe that this premise is also valid for PA programmes. Thus, it will help to improve services and, at the same time, to increase access and the level of PA of elderly citizens.

## Conclusions

Our findings suggest that although there are some good practices in the PA programmes under analysis, specifically in criteria *Processes*, *Leadership*, *Customer results *and *People*, there are still relevant areas that require improvement, namely those related to *Partnerships and resources*, *People results*, *Policy and strategy*, *Key performance results *and *Society results*.

### Strengths and Limitations

To our knowledge, this was the first study applying the EFQM Excellence Model criteria in PA programmes for elderly people.

However, the study has certain limitations, which must be considered when interpreting its results.

First, the study was based on the PA programmes coordinators' perceptions. Consequently, such perceptions may not provide a complete and accurate picture of the reality. Actually, the results are mainly based on self-reporting which might also have contributed to a more favourable outcome. Conducting a study with the participation of different stakeholders of the PA programmes will be an asset in the future. Secondly, the research design employed was cross-sectional rather than longitudinal. In this regard, an evaluation of the quality practices is a process that develops over time and whose effects are only really appreciated in the long term. Therefore, it would be appropriate to follow a longitudinal approach in future studies. Finally, the external validity of the findings presented is low. Nevertheless, we are convicted that the study provides details about the management models of the PA programmes for elderly people developed by the Portuguese Local Administration, their strengths and weaknesses, in order to improve their quality.

## Authors' contributions

AIM participated in the acquisition and analysis of data and participated in drafting and editing the manuscript. MJR managed the data collection and analysis and supervised the drafting and editing of manuscript. PS designed the study protocol and helped design the questionnaires/interviews. RS managed the data collection and analysis. JM participated in the coordination of the study and supervised the drafting and editing of manuscript. JC participated in the design of the questionnaires/interviews and coordination and management of the study.

All authors read and approved the final manuscript.

## Ethics approval

The study was approved by the Scientific Council and Ethics Committee of the Faculty of Sport - University of Porto.

## Competing interests

The authors declare that they have no competing interests.

## Pre-publication history

The pre-publication history for this paper can be accessed here:

http://www.biomedcentral.com/1471-2458/11/123/prepub

## Supplementary Material

Additional file 1 **Preliminary on-line questionnaire**. Explanation of the structure and content of the preliminary on-line questionnaire Click here for file
